# Stationsapotheker:innen in der Intensivmedizin: ökonomische Nutzenanalyse

**DOI:** 10.1007/s00063-023-01102-y

**Published:** 2024-01-23

**Authors:** Nadja Liebing, Benjamin Ziehr, Susanne Röber, Lutz Nibbe, Michael Oppert, Ulrich Warnke

**Affiliations:** 1 Apotheke, Klinikum Ernst von Bergmann gGmbH, Charlottenstr. 72, 14467 Potsdam, Deutschland; 2 Zentrum für Notfall- und Internistische Intensivmedizin, Klinikum Ernst von Bergmann gGmbH, Potsdam, Deutschland

**Keywords:** Klinisch-pharmazeutische Dienstleistungen, Arzneimitteltherapiesicherheit, Krankenhausapotheke, Pharmazeutische Interventionen, Ökonomischer Nutzen, Clinical pharmacy services, Medication safety, Hospital pharmacy, Pharmaceutical interventions, Economic benefit

## Abstract

**Hintergrund:**

Der positive Einfluss pharmazeutischer Betreuung auf die Verbesserung der Arzneimitteltherapiesicherheit gilt als belegt. Zum ökonomischen Nutzen klinisch pharmazeutischer Dienstleistungen in Deutschland ist bisher wenig bekannt.

**Ziel der Arbeit:**

Im Klinikum Ernst von Bergmann wurde 2020 ein Pilotprojekt zur Einführung von Stationsapotheker:innen in der Intensivmedizin gestartet, in dem auch der finanzielle Nutzen des angebotenen Medikationsmanagements ermittelt werden sollte.

**Methodik:**

Jeder pharmazeutischen Intervention (PI) wurde durch ein Team aus erfahrenen Intensivmediziner:innen und Stationsapotheker:innen im Konsensprinzip ein Wahrscheinlichkeitswert (Nesbit-probability-Score) zugeordnet, mit dem ein unerwünschtes Arzneimittelereignis (UAE) aufgetreten wäre. Unter der Annahme, dass pro UAE eine verlängerte Liegedauer resultiert, wurden die durchschnittlichen Fallkosten der Intensivstation/Tag als Einsparungspotenzial herangezogen. Das Modell kombiniert dabei die Ergebnisse zweier internationaler Publikationen, um eine ökonomische Bilanzierung pharmazeutischer Dienstleistungen zu ermöglichen.

**Ergebnisse:**

Im Untersuchungszeitraum wurden 177 PI ausgewertet und entsprechende Wahrscheinlichkeitswerte für das Eintreten von UAE ermittelt. Daraus wurden durch vermiedene Kosten jährliche Einsparungen von 80.000 € berechnet.

**Schlussfolgerung:**

In diesem Projekt konnte der ökonomische Nutzen pharmazeutischer Dienstleistungen in der Intensivmedizin belegt werden. Stationsapotheker:innen sind nun fester Bestandteil des intensivmedizinischen Behandlungsteams im Klinikum Ernst von Bergmann.

## Abstract (en)

**Background:**

The positive impact of pharmaceutical care in improving medication safety is considered proven. Little is known about the economic benefit of clinical pharmaceutical services in Germany.

**Objective:**

In 2020, a pilot project was started at the Ernst von Bergmann Hospital to introduce ward-based clinical pharmacists in intensive care medicine, also in order to determine the economic benefit of the medication management offered.

**Methods:**

By a team of experienced intensive care physicians and clinical pharmacists on the basis of a consensus principle, each pharmaceutical intervention (PI) was assigned a probability score (Nesbit probability score) with which an adverse drug event (ADE) would have occurred. Assuming that each ADE results in an increased length of stay, the costs of intensive care treatment/day were used as potential savings. The model thereby combines the findings of two international publications to enable an economic analysis of pharmaceutical services.

**Results:**

During the study period, 177 pharmaceutical interventions were evaluated and corresponding probability scores for the occurrence of ADE were determined. From this, annual savings of € 80,000 through avoided costs were calculated.

**Conclusion:**

In this project, the economic benefit of pharmaceutical services in intensive care medicine was proven. Ward-based clinical pharmacists are now an integral part of the intensive care treatment team at the Ernst von Bergmann Hospital.

Apothekerinnen und Apotheker auf der Station sind ein wesentlicher Bestandteil des „closed loop medication management“. Besonders in der Intensivmedizin können sie einen wichtigen Beitrag zur Verbesserung der Arzneimitteltherapiesicherheit leisten. Darum empfiehlt die Deutsche Interdisziplinäre Vereinigung für Intensiv- und Notfallmedizin e.V. (DIVI) bereits seit 2010, dass Stationsapotheker:innen feste Mitglieder im interprofessionellen Behandlungsteam auf der Intensivstation in der direkten Patientenversorgung sein sollen [29]. In diesem Beitrag wird ein Projekt zur Einführung dieser pharmazeutischen Dienstleistung sowie zur ökonomischen Nutzenanalyse vorgestellt.

## Einleitung

Ungefähr 95 von 100 Krankenhauspatient:innen erhalten zur Linderung oder Heilung ihrer Beschwerden medikamentöse Therapien. Allerdings ist Arzneimitteltherapie risikobehaftet und führt in Deutschland aufgrund von unerwünschten Arzneimittelwirkungen (UAW) zu über 5 % aller Krankenhausaufnahmen jährlich [28]. Auch während der Klinikaufenthalte treten arzneimittelbezogene Probleme (ABP) auf, von denen ein Großteil als vermeidbar angesehen wird [15, 18]. Diese umfassen neben UAW auch Medikationsfehler oder unerwünschte Arzneimittelereignisse (UAE). Eine vom Bundesgesundheitsministerium geförderte Studie hat ergeben, dass lediglich 29 % aller Nebenwirkungen bei bestimmungsgemäßem Gebrauch auftraten, also als unvermeidbar eingestuft werden können. Die übrigen 71 % müssen demnach als Folge von Medikationsfehlern angesehen werden. Die dadurch verursachten Kosten für das Gesundheitssystem sollen in Deutschland zwischen 800 Mio. und 1,2 Mrd. € jährlich betragen [9].

Die Aktionspläne des Bundesministeriums für Gesundheit (BMG) zur Verbesserung der Arzneimitteltherapiesicherheit (AMTS) tragen diesen Erkenntnissen seit nun mehr als 10 Jahren mit konkreten Maßnahmen Rechnung. Der Nutzen klinisch-pharmazeutischer Betreuung steht mittlerweile auch in Deutschland außer Frage.

Eine umfassende Auswertung der hierzulande in Krankenhäusern angebotenen pharmazeutischen Dienstleistungen wurde erstmals 2019 veröffentlicht [27]. Eine weitere Umfrage unter Intensivmediziner:innen ermittelte 11 unverzichtbare bzw. wünschenswerte Leistungen der Apotheke. Bei etwa einem Drittel der befragten 167 Intensivstationen war ein regelmäßiger pharmazeutischer Service bereits etabliert [11].

Während international der klinische und der finanzielle Vorteil von pharmazeutischer Betreuung stationärer Patient:innen nachgewiesen werden konnte [5, 7, 25], fehlen derzeit für Deutschland noch entsprechende aussagekräftige Analysen. Insbesondere über den ökonomischen Nutzen der pharmazeutischen Dienstleistungen in deutschen Krankenhäusern wurde bisher kaum publiziert.

Die Arzneimitteltherapie von kritisch kranken Patient:innen ist oft polypharmazeutisch sowie hoch komplex und deswegen besonders risikobehaftet. ABP können Therapieziele gefährden oder die Liegedauer verlängern. Die Integration von Stationsapotheker:innen in das intensivmedizinische Team kann die AMTS und damit die Patient:innensicherheit verbessern [5, 18–20, 30]. Zur Prävention von ABP sind in der Literatur verschiedene Maßnahmen beschrieben: regelmäßige Schulungen des ärztlichen und pflegerischen Personals, die Einführung von elektronischen Verordnungssystemen, die Festlegung von Standards sowie die Implementierung kontinuierlicher pharmazeutischer Betreuung, beispielsweise mittels Medikationsanalysen, Visitenbegleitung, Apothekenkonsilen oder Multimedikationsbesprechungen [3, 6].

Mit diesem Wissen wurde 2020 im Klinikum Ernst von Bergmann in Potsdam ein Pilotprojekt zur Einführung von Stationsapotheker:innen in der Intensivmedizin inklusive einer ökonomischen Nutzenanalyse gestartet. Ziel war es, mindestens die eingesetzten Personalkosten zu kompensieren. Mittlerweile liegen Daten für den Projektzeitraum von 24 Monaten vor, die wir hier berichten. Das Projekt ist abgeschlossen, wurde positiv bewertet und der pharmazeutische Betreuungsservice wurde in die Routine überführt.

## Methodik

Die pharmazeutischen Dienstleistungen, die in diesem Projekt etabliert wurden, umfassten unter anderem die analoge und digitale Kurvenvisite im Rahmen des klinischen Medikationsmanagements, interprofessionelle Visiten auf den Stationen und Mitarbeit bei der Erstellung von Therapiestandards, z. B. „Blutungsmanagement unter DOAK-Therapie“. Dabei wurden die anästhesiologische und die internistische Intensivstation sowie die Intermediate-Care-Station mit insgesamt 34 Betten und etwa 10.000 Belegungstagen/Jahr betreut. Die Fall- bzw. Liegedauerkosten betrugen im Durchschnitt 1480 €/Tag, ermittelt aus der kostenstellengerechten Buchung aller anfallenden Kosten pro Station. Um erweiterte Medikationsanalysen durchzuführen, arbeiteten die Stationsapotheker:innen den überwiegenden Teil des Projektzeitraums mit den auf den Intensivstationen befindlichen Papierkurven. Parallel begleiteten sie die Einführung des Patientendatenmanagementsystems Centricity^TM^ (High Acuity Critical Care 5.8, GE Healthcare GmbH, Solingen, Deutschland). Mit Überführung der vollständig integrierten elektronischen Patientenakte in die Routine war gegen Ende des Projekts auch die vorbereitende Kurvenvisite aus der Apotheke möglich. Für die Tätigkeiten wurden Personalkapazitäten in Höhe von 40 h/Woche eingesetzt. Es fanden 3‑mal pro Woche Visiten mit pharmazeutischer Beteiligung statt. Das interprofessionelle Visitenteam aus Chefarzt, Oberärzt:innen, stationsärztlichem Dienst und Stationsapotheker:innen wurde mindestens einmal wöchentlich durch Fachpersonal der Infektiologie, Mikrobiologie und Hygiene im Rahmen einer Antibiotic Stewardship-Visite verstärkt. Dabei wurden jeweils die bei der vorausgegangenen Medikationsanalyse identifizierten ABP diskutiert. ABP sind definitionsgemäß Ereignisse oder Umstände bei der Arzneimitteltherapie, die tatsächlich oder potenziell das Erreichen angestrebter Therapieziele verhindern. Dazu zählen neben den unerwünschten Arzneimittelwirkungen (UAW) auch unerwünschte Arzneimittelereignisse (UAE) sowie Medikationsfehler, die durch ein Abweichen von dem für die Patient:innen optimalen Medikationsprozess zu einer vermeidbaren Schädigung der Patient:innen führen können. Alle pharmazeutischen Interventionen wurden durch die Stationsapotheker:innen in einer Tabellenmatrix dokumentiert, die sich an der etablierten Form der ADKA-DokuPIK orientiert. DokuPIK steht für „Dokumentation pharmazeutischer Interventionen im Krankenhaus“ und ist eine Onlinedatenbank des Bundesverbands Deutscher Krankenhausapotheker e. V. (ADKA). Die Bewertung der ABP erfolgte zunächst mithilfe des 9‑stufigen NCC-MERP-Index (National Coordinating Council for Medication Error Reporting and Prevention) zur Kategorisierung von Medikationsfehlern, dessen Zuverlässigkeit zur Bewertung pharmazeutischer Interventionen nachgewiesen wurde [12]. Um die Primärdaten ökonomisch zu betrachten, wurde ein Modell entwickelt, das die Ergebnisse zweier vielzitierter Publikationen zusammenführt. Bates et al. (1997) bestimmten durch gepaarte Regressionsanalyse 247 unerwünschter Arzneimittelereignisse (UAE), dass sich die durchschnittliche Liegedauer durch ein UAE im Mittel um 2,2 Tage verlängert [2]. Da ABP aufgrund ihrer unterschiedlichen klinischen Relevanz nicht zwangsläufig zu einem UAE und damit einer Verlängerung der Liegedauer führen, wurde durch ein 4‑köpfiges Team aus Apotheker:innen und Intensivmediziner:innen im Konsensprinzip (Consensus Panel), wie von Nesbit et al. (2001) beschrieben, ein „Probability-Score“ bestimmt [23]. Dabei wurde jeder pharmazeutischen Intervention eine Wahrscheinlichkeit (*p*) zugeordnet, mit der ein UAE ohne diese Intervention aufgetreten wäre. Die Bewertung erfolgt nach der Einteilung, dass ein UAE mit keiner (*p* = 0), sehr geringer (*p* = 0,01), geringer (*p* = 0,1), mittlerer (*p* = 0,4) oder hoher (*p* = 0,6) Wahrscheinlichkeit eintritt. Die Summe (*N*) der einzelnen Wahrscheinlichkeiten (*p*) wird stellvertretend für die Anzahl an vermiedenen UAE in folgende Formel zur Berechnung der vermeidbaren Kosten (K_v_) eingefügt:

$$\begin{aligned}
\mathrm{Kv}&=N*L*\mathrm{KI}\\
N&=\sum p\left(\textit{Nesbit}\right)\ \left[UAE\right]\\
L&=\textit{Liegedauerverl{\"a}ngerung nach}\\
&\quad\textit{Bates}\ \left[\frac{\textit{Tag}}{UAE}\right]\\
KI&=\textit{Kosten Intensivbett}\ \left[\frac{\text{\EUR}}{\textit{Tag}}\right]
\end{aligned}$$

## Ergebnisse

Im Untersuchungszeitraum von 8 Monaten wurden innerhalb von 1741 eingeschlossenen Patiententagen durch die Stationsapotheker:innen insgesamt 177 Interventionen dokumentiert. Die häufigsten Gründe für Interventionen waren die Anpassung der Dosis (73 Interventionen), Arzneimittelinteraktionen (48 Interventionen) und Indikation/Arzneimittelauswahl (17 Interventionen; Tab. 1). Tab. 2 zeigt die Verteilung nach ATC-Klassifikation.

**Tab. 1:** Kategorisierung pharmazeutischer Interventionen nach Grund

Grund (Kategorie)	Anzahl an Interventionen (%)	
Dosis	73 (41,2)	
Interaktion	48 (27,1)	
Indikation/Arzneimittelauswahl	17 (9,6)	
Nebenwirkung	13 (7,3)	
Beratung von medizinischem Personal	9 (5,1)	
Anwendung	8 (4,5)	
Kontraindikation	5 (2,8)	
Doppelverordnung	2 (1,1)	
Beschaffung/Kosten	1 (0,6)	
Dokumentation/Übertragung	1 (0,6)	
Gesamt	177

**Tab. 2:** Kategorisierung pharmazeutischer Interventionen nach ATC-Klassifikation

		Pharmazeutische Interventionen		
ATC-Code	Bedeutung ATC-Code	Anzahl (%)	∑ Nesbit-Score	
J01	Antibiotika zur systemischen Anwendung	56 (31,6)	6,60	
A02	Antiepileptika	15 (8,5)	0,99	
N03	Mittel bei säurebedingten Erkrankungen	15 (8,5)	1,54	
N05	Psycholeptika	15 (8,5)	1,29	
N06	Psychoanaleptika	10 (5,6)	0,75	
C01	Herztherapie	8 (4,5)	0,85	
G04	Urologika	8 (4,5)	0,56	
B01	Antithrombotische Mittel	7 (4,0)	0,43	
–	Sonstige	43 (24,3)	3,12	
Gesamt		177	16,13	

Die Verteilung der Probability-Scores der insgesamt 177 Interventionen stellt sich zusammengefasst wie folgt dar: Etwas weniger als die Hälfte nehmen geringe und mittlere Wahrscheinlichkeiten eines UAE ein (67 bzw. 20 Interventionen; 37,9 % bzw. 11,3 %). Lediglich eine Intervention war mit einer hohen Wahrscheinlichkeit für ein UAE verknüpft (0,6 %). Die Anzahl aller Interventionen multipliziert mit ihrer zugehörigen Wahrscheinlichkeit beträgt in Summe 16,13. Daraus folgt, dass durch die pharmazeutischen Interventionen 16,13 UAE und somit eine potenzielle Liegedauerverlängerung von 35,49 Tagen verhindert werden konnten (Tab. 3).

$$\mathrm{Kv}=N*L*\mathrm{KI}$$

$$\mathrm{Kv}=16.13\left[UAE\right]*2{,}2\,\left[\frac{Tage}{UAE}\right]*1480\left[\frac{\text{\EUR}}{Tag}\right]$$

$$\mathrm{Kv}=52.519{,}28\,\text{\EUR}$$

**Tab. 3:** Anzahl und Verteilung der Wahrscheinlichkeiten

	Pharmazeutische Interventionen		
Wahrscheinlichkeit UAE (Nesbit-probability-Score)	Anzahl (%)	∑ Nesbit-Score	Vermiedene Kosten, €
Null (0)	6 (3,4)	0	0
Sehr gering (0,01)	83 (46,9)	0,83	2702,48
Gering (0,1)	67 (37,9)	6,7	21.815,20
Mittel (0,4)	20 (11,3)	8,0	26.048,00
Hoch (0,6)	1 (0,6)	0,6	1953,60
Gesamt	177	16,13	52.519,28

Durch die pharmazeutische Betreuung resultierten im Untersuchungszeitraum Kosteneinsparungen in Höhe von 52.519,28 €. Extrapoliert man die Daten auf den Zeitraum eines Jahrs, ergibt sich eine Gesamtersparnis von circa 80.000 €. Damit wurde das angestrebte Ziel erreicht.

## Diskussion

**Abb. 1:**
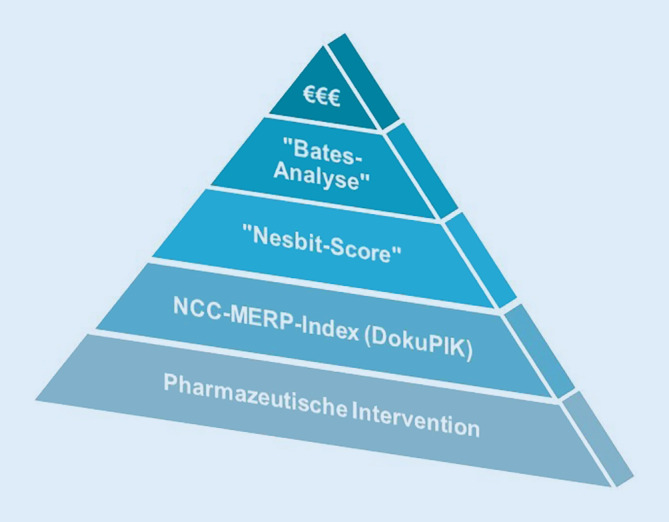
Pharmakoökonomisches Modell, schematisch

Zur Entwicklung der Methode und zur Ermittlung der Ergebnisse wurden Modelle aus 2 unabhängig voneinander publizierten Arbeiten kombiniert, wodurch die Abschätzung des ökonomischen Nutzens pharmazeutischer Arbeit möglich wird (Abb. 1). Wegen der kontinuierlichen Intervention der Stationsapotheker:innen im gesamten Projektzeitraum können keine Daten im Sinne einer Kontrollphase ohne pharmazeutische Betreuung präsentiert werden. In der Zukunft sind deshalb weitergehende Untersuchungen zur Validierung der Methodik notwendig.

Berücksichtigt man die anteiligen Personalkosten der Stationsapotheker:innen im Projekt, die auf die Identifizierung und Besprechung von PI entfallen, nämlich 33.800 €, ergäbe sich eine „bereinigte“ Einsparung von 46.200 € pro Jahr. Hochgerechnet würde sich modellhaft bei ausschließlicher Durchführung von PI als Betreuungsleistung die Einsparung auf 102.800 € p. a. mehr als verdoppeln.

Bemerkenswert ist, dass eine Wiederholungsuntersuchung innerhalb des 24-monatigen Projektzeitraums, die noch nicht mittels Konsensprinzip bewertet wurde, ein vergleichbares Ergebnis (Nesbit-Score 12,57; Zeitraum 5 Monate; Einsparung circa 98.000 € p. a., bereinigt 142.800 € p. a.) zeigen konnte.

Die Wahrscheinlichkeit, dass ein UAE ohne Intervention stattfindet, wird im vorliegenden Modell mit maximal 60 % (*p* = 0,6) bewertet, obwohl die Wahrscheinlichkeit im Einzelfall deutlich höher liegen kann. Um die klinische Relevanz der Interventionen stärker in den Fokus zu nehmen, wurde das Consensus Panel im Unterschied zu Nesbit et al. durch ärztliches Personal erweitert. Diese Maßnahme scheint potenzielle Bestätigungsfehler (englisch: „confirmation bias“) zu minimieren, da die Summe der ausschließlich von Apotheker:innen bewerteten Wahrscheinlichkeitsscores etwa um ein Drittel höher lag (N = 25,81 vs. N = 16,13).

Pharmazeutische Interventionen verbessern die AMTS und haben einen positiven Einfluss auf das klinische Ergebnis von Krankenhauspatient:innen [1, 8, 13]. Aufgrund der vielfältigen pharmazeutischen Aufgaben in diesem Projekt liegen die in die Auswertung eingeschlossenen PI mit einer Rate von 7,7 pro 100 Patiententagen im Vergleich mit anderen Untersuchungen etwas niedriger [17]. Zusätzlich waren die Vorteile einer elektronischen Patientenakte erst am Ende des Projektzeitraums nutzbar.

Anzumerken ist, dass die Bewertung des ökonomischen Nutzens der pharmazeutischen Betreuung konservativ erfolgte. Das Modell ermittelte ausschließlich die durch eine etwaige um 2,2 Tage verlängerte Liegedauer verursachten Kosten und lässt weitere Einsparungseffekte außer Acht. Beispielsweise wurden im Projektzeitraum zusätzlich direkte Kosteneinsparungen in Höhe von über 15.000 € durch die ökonomische Optimierung der Pharmakotherapie erzielt. Diese Einsparungen wurden im beschriebenen Modell nicht berücksichtigt. Vergleichbare direkte Kosteneinsparungen konnten aber bereits in anderen Untersuchungen nachgewiesen werden [14, 21]. In einer weiteren Studie aus dem Jahr 2012, die Patient:innen verschiedener Fachdisziplinen aus 3 deutschen Krankenhäusern einschloss, wurden insbesondere die ökonomischen Konsequenzen von UAW untersucht [26]. Die Autoren ermittelten für Patient:innen mit UAW um 970 € höhere Behandlungskosten und eine im Durchschnitt sogar um 2,9 Tage längere Liegedauer.

Obwohl es offensichtlich ist, dass durch pharmazeutische Betreuung die AMTS erhöht werden kann, wurde dies hier nicht explizit untersucht. Dennoch wurde bereits in der Vergangenheit nachgewiesen, dass die direkte Beteiligung von Apotheker:innen am Medikationsprozess in Krankenhäusern ABP aufdecken und Risiken minimieren kann [3, 4, 10, 22, 24]. Zum ökonomischen Nutzen dieser Beteiligung in deutschen Krankenhäusern gibt es bisher allerdings kaum Daten. Dies könnte eine Ursache dafür sein, dass die Zusammenarbeit von Ärzt:innen und Apotheker:innen in der Intensivmedizin, so wie im Übrigen seit 2010 und jüngst aktualisiert von der Deutschen Interdisziplinären Vereinigung für Intensiv- und Notfallmedizin (DIVI) e. V. gefordert [29], in Deutschland erst ansatzweise umgesetzt ist. Dass die Beteiligung von Stationsapotheker:innen durchaus auch kostendämpfende Effekte hat und sich sogar mindestens gegenfinanziert, konnte für die Intensivmedizin durch das hier beschriebene Projekt gezeigt werden. Die Übertragbarkeit in andere medizinische Fachdisziplinen sollte weiter untersucht werden.

Für eine nachhaltige und flächendeckende Überführung in die klinische Routine allerdings bedarf es kreativer Finanzierungskonzepte. Die Aussagen von Klinikgeschäftsführern unterstreichen das: Stationsapotheker:innen seien aus pharmakologischer Sicht absolut wünschenswert, denn durch sie würde die maximale Kompetenz im Hinblick auf Arzneimittel und deren Wirkung direkt vor Ort am Patienten verfügbar sein. „Da das Gesundheitswesen aber eine sehr knappe Finanzierung vorsieht, müsste ein solcher Einsatz von zusätzlichem Personal zwingend auch wirtschaftlich darstellbar sein. Wenn es eine Finanzierung für die Vorhaltung zusätzlicher Apotheker gäbe, würde dies sicher zu einer verbesserten Arzneimitteltherapie am Punkt der Versorgung führen“ [16].

Der Bundesverband der Deutschen Krankenhausapotheker ADKA e. V. sieht verschiedene Modelle: Innerhalb des regulatorischen Rahmens könnte das Stationsapotheker:innenkonzept in entsprechende Gesetze aufgenommen werden, wie das Beispiel Niedersachsen zeigt. Am vielversprechendsten scheint aber im Moment der Ansatz der Aufnahme in eine Qualitätssicherungsrichtlinie des G‑BA mit festgelegten Strukturkriterien. Die Finanzierung würde dann im DRG-System Berücksichtigung finden. Ein anderer Weg wäre die Integration klinisch-pharmazeutischer Dienstleistungen in die Vorhaltepauschale von Krankenhäusern. Hier bleibt die weitere Entwicklung bei der Umsetzung der Empfehlungen der Regierungskommission für eine moderne und bedarfsgerechte Krankenhausversorgung abzuwarten.

Im ambulanten Sektor wurde Ende 2020 mit dem Vor-Ort-Apotheken-Stärkungsgesetz (VOASG) der Anspruch der Patient:innen auf pharmazeutische Dienstleistungen und deren Honorierung gesetzlich festgeschrieben. Die Dienstleistungen umfassen insbesondere Maßnahmen der Apotheken zur Verbesserung der Sicherheit und Wirksamkeit einer Arzneimitteltherapie (§ 129 Abs. 5e SGB V). Damit ist für öffentliche Apotheken erstmalig eine gesetzlich geregelte Finanzierung für die Durchführung von pharmazeutischen Dienstleistungen vorhanden.

Daraus ergibt sich die Frage, ob dieses Modell nicht auch in den stationären Sektor übertragbar wäre, denn insbesondere in der Intensivmedizin kann pharmazeutische Betreuung das Ziel der Verbesserung der Sicherheit und Wirksamkeit von Arzneimitteltherapie erreichen und, wie hier gezeigt, einen ökonomischen Nutzen darstellen. Von diesen Vorteilen profitieren zuallererst die Patient:innen, aber auch das therapeutische Team und nicht zuletzt die Kostenträger.

## Fazit für die Praxis

- Stationsapotheker:innen im intensivmedizinischen Team vermeiden zusätzliche Behandlungskosten.
- Die Ergebnisse unterstützen die aktuellen DIVI-Empfehlungen zur Struktur und Ausstattung von Intensivstationen mit Stationsapotheker:innen.
- Zur umfassenden Etablierung in deutschen Krankenhäusern werden Umsetzungs- und insbesondere Finanzierungskonzepte benötigt.
